# A rare complication of continuous positive airway pressure treatment – rectus sheath hematoma: a case report

**DOI:** 10.1016/j.bjane.2021.02.039

**Published:** 2021-03-22

**Authors:** Adnan Yamanoğlu, Serkan Bilgin, Nalan Gokce Celebi Yamanoğlu, Fatih Esad Topal

**Affiliations:** a Ataturk Training and Research Hospital, Izmir Katip Celebi University, Department of Emergency Medicine, Izmir, Turkey; b Izmir Bozyaka Training and Research Hospital, Department of Emergency Medicine, Izmir, Turkey

**Keywords:** Bedside ultrasound, Chronic obstructive pulmonary disease, Dyspnea, Emergency medicine, Noninvasive mechanical ventilation

## Abstract

Noninvasive mechanical ventilation (NIMV) has a relevant role in the treatment of critically ill patients displaying severe dyspnea. Continuous positive airway pressure (CPAP), a method of NIMV, is also widely used in the management of acute heart failure, chronic obstructive pulmonary disease (COPD) exacerbation, and symptomatic sleep apnea. However, numerous traumatic complications of CPAP treatment in the face region, head, and thorax have been reported and may be related to the application of a continuous positive high pressure to the airway. Conversely, we have observed no complications due to CPAP-related increased intra-abdominal pressure. In this article, we describe a clinical case of a patient with an acute rectus sheath hematoma during CPAP treatment. This previously unreported complication demonstrates that CPAP should be carefully used in patients with exacerbated COPD with difficulty in expiration

## Introduction

The continuous positive airway pressure (CPAP) device, first described by Sullivan et al. in 1981, produces continuous positive pressure due to its high-speed motor, and successfully keeps the upper respiratory tract open.^1^ CPAP treatment has a wide sphere of use, particularly in sleep apnea syndrome, as well as acute cardiogenic pulmonary edema (ACPE), chronic obstructive pulmonary disease (COPD), postsurgical lung-protective procedures, and viral bronchiolitis.^2^

Various trauma-related adverse effects resulting from the high pressure to which patients are exposed may be seen in CPAP treatment. CPAP has previously been reported to be capable of causing maxilla-facial injuries, increased intraocular pressure and tympanic membrane rupture associated with high pressure developing in the face;^3^ pneumothorax-pneumomediastinum and lung herniation associated with high pressure in the thorax;^4^ and pneumocephalus associated with increased intracranial pressure. However, we have encountered no previously reported complication developing in association with increased intra-abdominal pressure in CPAP treatment.

This report describes a case of sudden-onset rectus sheath hematoma (RSH) with no provoking factor other than CPAP treatment in a 66-year-old male patient.

## Case report

A 66-year-old man presented to the emergency department (ED) with shortness of breath. Diagnoses of COPD and heart failure (HF) were present in his medical history. The patient was taking ipratropium bromide monohydrate plus salbutamol sulfate chronically for COPD, and budesonide in addition to home treatment during attacks. Additionally, he was taking valsartan and hydrochlorothiazide chronically for HF. The patient's respiration rate was 22 with pronounced abdominal breathing, while other vital parameters were within normal ranges, and oxygen saturation was 91%. A physical examination revealed bilateral basal end-inspiratory crackles and rhonchus during the expiratory phase, with prolonged expiration. Cardiomegaly was determined in bedside chest X-ray. CPAP was initiated at a setting of 5 cm H2O external positive end-expiratory pressure (PEEP) via a mask covering the mouth and nose with preliminary diagnoses of COPD exacerbation and HF. No accompanying cough was present, and no significant problems were experienced in terms of CPAP therapy compliance; however, a previously non-existing abdominal pain commenced at approximately the first hour of treatment.

**Figure 1:**
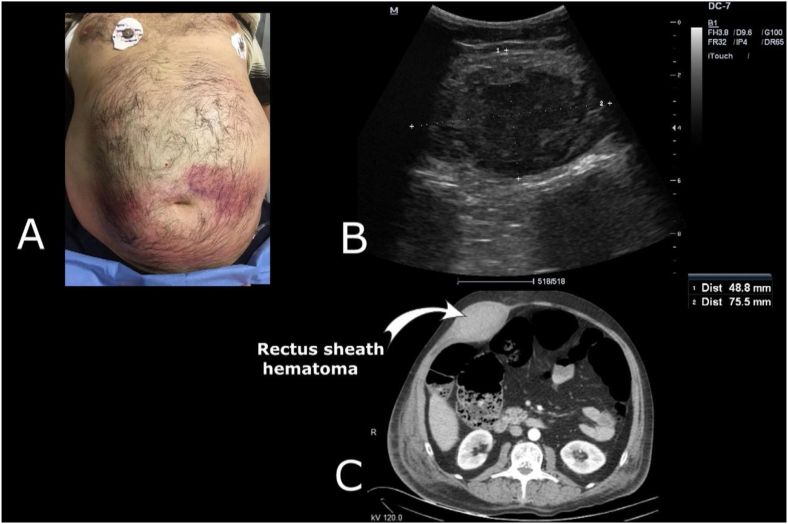
Physical examination finding, ultrasound image, and CT image of the patient with rectus sheath hematoma. A, Redness and ecchymotic areas of abdominal skin that occurred within minutes after abdominal pain; B, Ultrasonographic measurement of the diameter of rectus sheath hematoma with curvilinear probe; C, Computed tomography image showing rectus sheath hematoma that did not invade into the intra-abdominal area.

The abdominal pain was exacerbated when the patient was in the expiratory phase and the patient was unable to continue CPAP treatment. Tenderness was present around the umbilical region in an initial examination, and redness and ecchymotic areas around the umbilical region and extending to the lower quadrants appeared within minutes (Fig. 1A). Pancreatic enzymes were requested due to the similarity to Cullen's sign, and bedside ultrasound (USG) was performed. Rapid USG revealed no intra-abdominal free fluid. However, a suspected echogenicity was detected inside the abdominal rectus muscles. The rectus muscle was examined in detail using a curvilinear and linear probe, and a 75*48 mm-diameter hematoma was detected inside the rectus muscle (Fig. 1B). Laboratory tests revealed white blood cells (WBC): 12.53 (10^3^uL); platelets: 383 (10^3^uL); partial thromboplastin time (PTT): 12.006 (s); activated PTT 23.785 (s); International Normalized Ratio (INR): 1.017; amylase: 64 (U.L^-1^); and lipase: 11(U.L^-1^). No bleeding disorder or additional intra-abdominal pathology was determined, and abdominal computed tomography (CT) was performed to confirm the diagnosis of RSH and to confirm the requirement for surgery. The RSH did not open into the abdomen, and no intra-abdominal organ pathology was detected (Fig. 1C).

The patient was hospitalized for 7 days with a diagnosis of HF and COPD exacerbation. During the hospital stay, the patient was given respiratory support with only high-flow oxygen for the first 24 hours. For the next 3 days, bi-level positive airway pressure (BPAP) and alternating breathing support with high-flow oxygen were administered. The medications of the patient, who did not need respiratory support except nasal oxygen therapy for the previous 3 days, were rearranged. The patient, who did not progress in terms of the RSH, which partially resorbed by the seventh day (70*45 mm), was discharged without undergoing surgery with the recommendation of conservative follow-up.

## Discussion

RSH is characterized by an accumulation of blood within the rectus sheath due to a hemorrhage originating from branches of the inferior epigastric artery at its insertion into the rectus abdominis muscle, where branches of the lower epigastric artery are often the most vulnerable. Although RSH is a rare clinical condition, it is more common in elderly and female patients, and the most significant risk has been linked to trauma and anticoagulant therapy.^5^ Atherosclerosis, hypertension, cirrhosis, and renal diseases can also increase the risk of rectus sheath hematoma,^5^ and coughing episodes in COPD and asthma can also constitute a risk by increasing intra-abdominal pressure. Of these risk factors, trauma and anticoagulant therapy were not present in our case, and values for coagulation tests were within normal limits. No cough capable of increasing intra-abdominal pressure accompanying the clinical manifestation was also present before or during ED follow-up.

CPAP therapy is primarily recommended as a noninvasive mechanical ventilation therapy in ACPE, and BPAP is primarily recommended in acute exacerbations of COPD, although many studies have shown that CPAP might be useful in exacerbations of COPD.^2^ CPAP may be particularly suitable for patients with COPD who find bi-level NIV uncomfortable, or who are not synchronized with the ventilator. However, the priority of treatment should be determined according to the clinic in patients with both diseases, like our patient.

Patients undergoing CPAP therapy should be closely monitored for both their underlying diseases and CPAP complications. It has been previously reported that CPAP might increase intracranial^3^ and intra-thoracic pressure,^4^ causing various complications related to trauma. However, no complications related to the abdomen have been reported by increasing intra-abdominal pressure. During the CPAP treatment of our patient, sudden onset abdominal pain developed with no cough or similar maneuver capable of increasing intra-abdominal pressure. During COPD acute exacerbations, scalene and sternocleidomastoid muscles are used more as accessory muscles in inspiration, and the abdominal respiratory muscles (rectus abdominis, transverse abdominis, and external and internal obliques) are used as accessory muscles in expiration. For this reason, the abdominal respiratory muscles used in expiration during an attack are already under additional pressure load. Also, we think that CPAP treatment could increase the difficulty of expiration in a patient who has an acute exacerbation of COPD. The patient may, therefore, have had to make greater use of the abdominal muscles during expiration. CPAP treatment, which makes expiration difficult for patients with COPD using abdominal muscles due to respiratory difficulties, may increase intra-abdominal pressure and cause rectus sheath hematoma.

## Conclusion

CPAP treatment may lead to rectus sheath hematoma by increasing intra-abdominal pressure in patients with COPD with difficulty in expiration. These patients may require attention during CPAP treatment or may be given priority to biphasic noninvasive ventilation methods rather than continuous methods.

## Conflicts of interest

The authors declare no conflicts of interest.
